# Increase in Colorado Tick Fever Virus Disease Cases and Effect of COVID-19 Pandemic on Behaviors and Testing Practices, Montana, 2020

**DOI:** 10.3201/eid2903.221240

**Published:** 2023-03

**Authors:** Raymond A. Soto, Erika Baldry, Grace M. Vahey, Jennifer Lehman, Margaret Silver, Amanda Panella, Aaron C. Brault, Holly R. Hughes, Kelly A. Fitzpatrick, Jason Velez, Brad J. Biggerstaff, Brent Wolff, Jean Randolph, Laird J. Ruth, J. Erin Staples, Carolyn V. Gould

**Affiliations:** Centers for Disease Control and Prevention, Fort Collins, Colorado, USA (R.A. Soto, G.M. Vahey, J. Lehman, M. Silver, A. Panella, A.C. Brault, H.R. Hughes, K.A. Fitzpatrick, J. Velez, B.J. Biggerstaff, J.E. Staples, C.V. Gould);; Montana Department of Health and Human Services, Helena, Montana, USA (E. Baldry);; Centers for Disease Control and Prevention, Atlanta, Georgia, USA (B. Wolff, J. Randolph, L.J. Ruth)

**Keywords:** Colorado tick fever virus, tickborne disease, COVID-19, coronavirus disease, SARS-CoV-2, severe acute respiratory syndrome coronavirus 2, viruses, respiratory infections, zoonoses, vaccine-preventable diseases, PCR testing, Montana, vector-borne infections, United States

## Abstract

In 2020, Montana, USA, reported a large increase in Colorado tick fever (CTF) cases. To investigate potential causes of the increase, we conducted a case–control study of Montana residents who tested positive or negative for CTF during 2020, assessed healthcare providers’ CTF awareness and testing practices, and reviewed CTF testing methods. Case-patients reported more time recreating outdoors on weekends, and all reported finding a tick on themselves before illness. No consistent changes were identified in provider practices. Previously, only CTF serologic testing was used in Montana. In 2020, because of SARS-CoV-2 testing needs, the state laboratory sent specimens for CTF testing to the Centers for Disease Control and Prevention, where more sensitive molecular methods are used. This change in testing probably increased the number of CTF cases detected. Molecular testing is optimal for CTF diagnosis during acute illness. Tick bite prevention measures should continue to be advised for persons doing outdoor activities.

Colorado tick fever (CTF) virus is a coltivirus in the family *Reoviridae* (*1*). The primary vector for CTF virus is the Rocky Mountain wood tick (*Dermacentor andersoni*), which is found at elevations of 4,000–10,000 feet in the western United States and Canada (*2*,*3*). The incubation period of CTF virus is usually 3–4 days (range 1–14 days). Patients with CTF virus disease commonly experience fever, headache, fatigue, myalgias, and a biphasic course (i.e., remission and relapse of symptoms with 1–4 days between remission and relapse). Approximately 15%–30% of patients with CTF are hospitalized, but severe complications such as meningoencephalitis, hepatitis, and epididymoorchitis are uncommon, occurring in <5% of patients; deaths related to CTF are rare (<1%) (*4*–*7*).

CTF virus disease is not nationally notifiable but is reportable in 9 states (Arizona, Colorado, Idaho, Montana, New Mexico, Oregon, South Dakota, Utah, and Wyoming), which voluntarily report cases to the Centers for Disease Control and Prevention (CDC). Because of shifts in reporting and testing practices, the numbers of cases reported from various states has fluctuated over time. During 2002–2019, a total of 108 CTF cases were identified from western states, a median of 5 cases per year, and <1 case per year in Montana (*7*,*8*). During 2020, a total of 21 CTF cases were reported among Montana residents.

The COVID-19 pandemic had several potential effects on risk, detection, and reporting of vectorborne diseases, including changes in individual behaviors affecting vector exposures, healthcare-seeking behaviors, healthcare provider test ordering practices, and diagnostic testing procedures, because of increased burden on state laboratories for SARS-CoV-2 testing. For example, CDC guidance recommended that persons engage in outdoor recreation to remain active while maintaining social distancing and reducing transmission of SARS-CoV-2 (*9*). In 2020, a historic number of persons visited national forests for recreation (*10*). 

Our aim was to investigate the increase in CTF cases in Montana in 2020, including the potential effect of the COVID-19 pandemic and risk factors for CTF virus disease among residents of Montana. Therefore, we conducted a case–control study, surveyed healthcare providers, and evaluated diagnostic testing practices for CTF virus infection.

## Methods

### Case–Control Study

Testing for CTF virus infection at CDC’s Arbovirus Diagnostic Laboratory (Fort Collins, CO, USA; Division of Vector-Borne Diseases, National Center for Emerging and Zoonotic Infectious Diseases) is performed at the request of state health departments. Testing uses reverse transcription PCR (RT-PCR) on serum or cerebrospinal fluid (CSF) samples collected <14 days after symptom onset and plaque-reduction neutralization test (PRNT) on specimens collected >14 days after symptom onset, as previously described (*11*,*12*). During the period under investigation, we conducted RT-PCR testing using primers targeting segment 3 of the viral genome (*12*). We based neutralizing antibody titers on the highest dilution of serum that reduced viral plaque formation by >90% (we considered a titer >10 to be positive). For specimens collected 8–21 days after symptom onset, we tested using both RT-PCR and PRNT on a case-by-case basis. We defined laboratory-confirmed recent infection as detection of CTF viral nucleic acid in a specimen or a seroconversion with >4-fold change in virus-specific neutralizing antibody titers between paired acute and convalescent serum specimens. We defined a probable infection as detection of virus-specific neutralizing antibodies in a single specimen because the timing of infection cannot be determined. IgM testing typically is not performed because the sensitivity for detection of IgM is lower than for neutralizing antibodies (*11*).

We identified case-patients and controls through a query of the Arbovirus Diagnostic Laboratory database. We defined a case-patient as a Montana resident who had symptom onset and a confirmed or probable CTF virus infection in 2020. We defined a control as a Montana resident who had symptom onset but negative testing for CTF virus infection in 2020. We contacted persons by phone to describe the investigation and offer participation in a survey collecting data on demographics, clinical symptoms, outdoor recreational and occupational exposures, tick exposures and prevention measures, and changes in behavior related to the COVID-19 pandemic. We collected specific location data for recreational activities and tick exposures whenever possible. Survey questions focused on the potential incubation or 2-week period before symptom onset in 2020 and the same period in 2019.

### Healthcare Provider Survey

We developed a survey for healthcare providers and distributed them to staff in 13 hospitals in 10 public health jurisdictions in Montana where either a CTF case was reported in the previous 10 years or CTF virus testing was requested in 2020. The survey collected information on healthcare provider type and demographics, awareness and testing practices for CTF virus and other tickborne diseases, patient encounters for tick bites, and healthcare providers’ interest in educational resources for CTF.

### Diagnostic Testing Evaluation

We reviewed diagnostic laboratory methods used for CTF testing during 2020 and in previous years and calculated the proportion of specimens testing positive. To assess whether other tickborne diseases transmitted by *Dermacentor andersoni* ticks increased in Montana during 2020, we also examined trends in reported cases of Rocky Mountain spotted fever (RMSF) and tularemia.

### Data Analysis

We managed data from participant interviews and the healthcare provider survey in Research Electronic Data Capture (Vanderbilt University, https://www.project-redcap.org) (*13*) and analyzed data using SAS (SAS Institute Inc., https://www.sas.com). For categorical variables, we calculated 95% CIs of the odds ratios (ORs) between case-patients and controls by using the score interval (*14*); given the rarity of CTF in the population, we expected OR estimates to provide reliable estimates of relative risk. For quantitative variables, we calculated 95% CIs of the differences in means by using the Welch-Satterthwaite approximation for the Student *t* interval (*15*). We determined statistical significance from the reported 95% CIs by noting whether they contained the null values of 1 for the ORs and 0 for the mean differences, enabling interpretation in a hypothesis-testing context at a significance level of α = 0.05. We performed geospatial mapping of tick exposure locations by using ArcGIS 10.7.1 (Environmental Systems Research Institute, https://www.esri.com).

## Results

### Case–Control Study

Of 107 potential participants identified, including 21 case-patients and 86 controls, we were able to contact 36 (14 [67%] case-patients and 22 [26%] controls) who agreed to participate. Both groups consisted of predominantly non-Hispanic White men and had similar age distributions. Most participants from both groups reported having no underlying medical conditions. About one third of participants reported being tested for SARS-CoV-2 when they sought healthcare for their symptoms, and all reported a negative test result (Table 1).

**Table 1:** Demographic characteristics of participants in a Colorado tick fever case–control study, Montana, USA, 2020*

Characteristic	Case-patients, n = 14	Controls, n = 22	OR (95% CI)
Sex			
M	10 (71)	12 (55)	2.1 (0.5–8.3)
F	4 (29)	10 (45)	0.5 (0.1–2.0)
Age group, y			
<21	5 (36)	4 (18)	2.5 (0.6–11.0)
21–44	3 (21)	10 (45)	0.3 (0.1–1.4)
45–64	5 (36)	5 (23)	1.9 (0.4–7.9)
>65	1 (7)	3 (14)	0.5 (0.1–3.9)
Median age, y (range)	26 (1–70)	33 (7–84)	NA
Race			
White	14 (100)	22 (100)	0.6 (0.4–11.7)
Ethnicity			
Non-Hispanic or non-Latino	12 (86)	17 (77)	1.8 (0.3–9.2)
Hispanic or Latino	1 (7)	3 (14)	0.5 (0.1–3.9)
Other	1 (7)	2 (9)	0.8 (0.1–6.7)
Underlying medical condition			
None	11 (79)	13 (59)	2.5 (0.6–10.8)
Immunosuppressive condition or medication	0	2 (9)	0.3 (0.0–3.6)
Diabetes mellitus	0	2 (9)	0.3 (0.0–3.6)
Cardiovascular disease	1 (7)	3 (14)	0.5 (0.1–3.9)
Cancer	0	1 (5)	0.1 (0.0–1.3)
Other	2 (14)	2 (9)	1.7 (0.3–10.9)
SARS-CoV-2 testing during illness			
Tested	6 (43)	7 (32)	1.6 (0.4–6.3)
Positive	0	0	1.6 (0.1–28.1)

*Values are no (%) except as indicated. NA, not applicable; OR, odds ratio.

**Figure 1:**
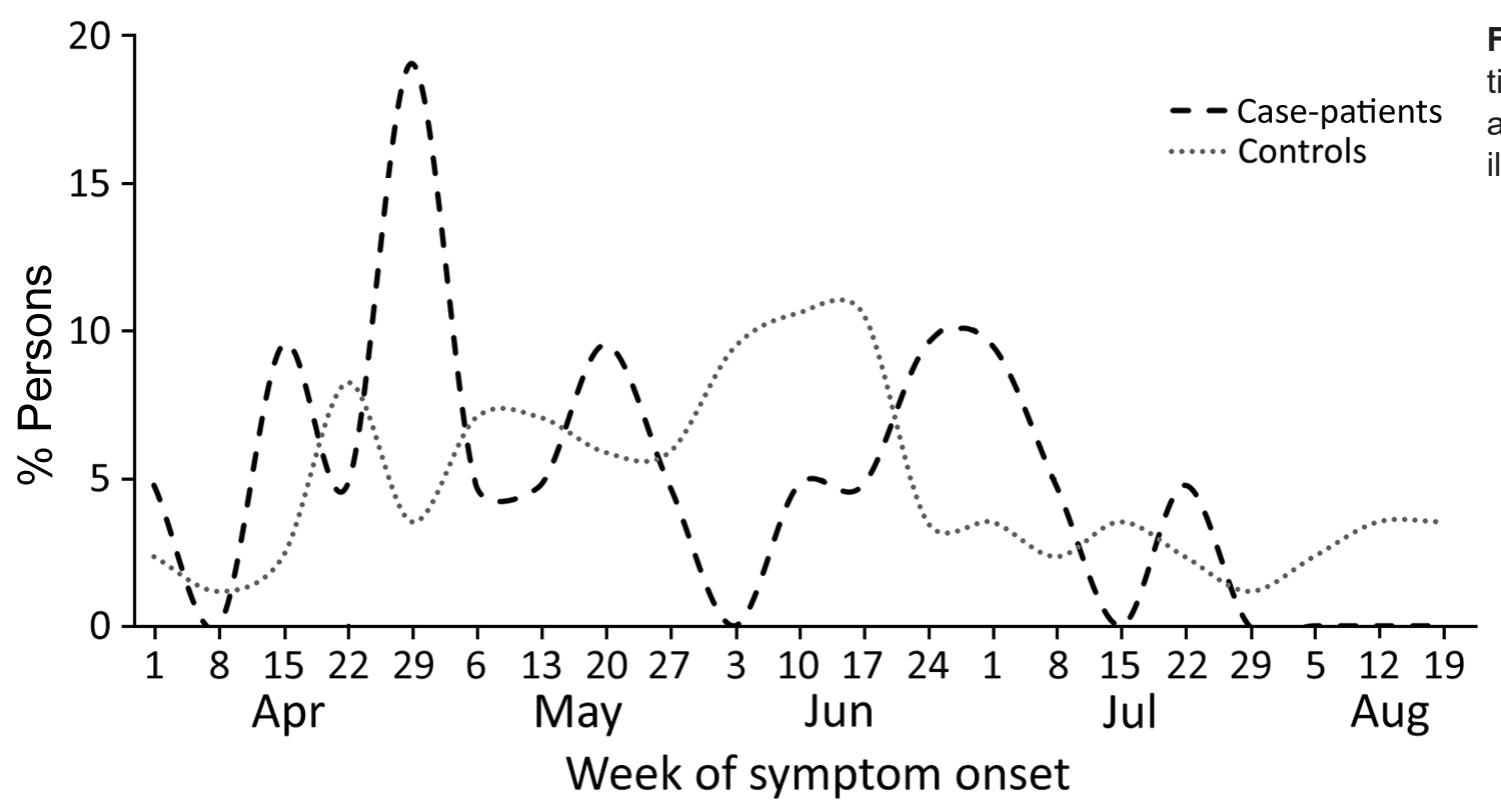
Percentage of Colorado tick fever case-patients (n = 21) and controls (n = 86), by week of illness onset, Montana, USA, 2020.

Symptom onset dates for case-patients occurred during April 1–July 31, 2020; for controls, symptoms occurred during April 1–August 22, 2020. Case-patients were more likely to become symptomatic earlier in the year, having a peak in late April, compared with controls (Figure 1). Symptoms reported by over half of the participants were similar between groups and included fever, fatigue, muscle aches, headache, chills, weakness, and joint pain and swelling (Table 2). Case-patients (11 [79%]) were statistically significantly more likely to report a biphasic illness than controls (3 [14%]). 

**Table 2:** Self-reported symptoms leading to Colorado tick fever virus testing of case–control study participants, Montana, USA, 2020*

Symptom	No. (%)		OR (95% CI)
	Case-patients, n = 14	Controls, n = 22	
Fever	13 (93)	14 (64)	7.4 (1.0–50.7)
Fatigue	13 (93)	18 (82)	2.9 (0.4–21.0)
Muscle aches	12 (86)	18 (82)	1.3 (0.2–7.2)
Headache	11 (79)	17 (77)	1.1 (0.2–4.9)
Chills	9 (64)	13 (59)	1.3 (0.3–4.8)
Weakness	9 (64)	17 (77)	0.5 (0.1–2.2)
Nausea	8 (57)	9 (41)	1.9 (0.5–7.3)
Joint pain or swelling	8 (57)	15 (68)	0.6 (0.2–2.4)
Rash	5 (36)	13 (59)	0.4 (0,1–1.5)
Stiff neck	5 (36)	10 (45)	0.7 (0.2–2.6)
Abdominal pain	5 (36)	5 (23)	1.9 (0.4–7.9)
Swollen lymph nodes	4 (28)	11 (50)	0.4 (0.1–1.5)
Confusion	4 (28)	10 (45)	0.5 (0.1–1.9)
Vomiting	4 (28)	3 (14)	2.5 (0.5–12.3)
Diarrhea	3 (21)	5 (23)	0.9 (0.2–4.4)
Sore throat	3 (21)	7 (32)	0.6 (0.1–2.6)
Biphasic illness†	11 (79)	3 (14)	**23.2 (4.2–128.6)**

*Bold indicates statistical significance (α = 0.05). OR, odds ratio. †Defined as remission and relapse of symptoms with 1–4 d between remission and relapse when dates were provided.

Almost half of all participants (44% [16/36]) reported spending more time outside in 2020 because of the COVID-19 pandemic compared with the same period in 2019, and we identified no difference between groups. A statistically significantly greater proportion of case-patients (86%) reported spending >4 hours outdoors recreating on weekends than controls (50%) (Table 3). Participants reported several outdoor activities; hiking or walking on unpaved trails was most common for both groups. Case-patients reported fewer instances of yardwork or gardening in the 2-week period before symptom onset than did controls and were statistically significantly more likely than controls to report finding a tick crawling on themselves (100% vs. 41%) or a tick attached to themselves (93% vs. 36%) (Table 3).

**Table 3:** Outdoor and tick exposures during the 2 weeks before illness onset for participants in a Colorado tick fever case–control study, Montana, USA, 2020*

Risk factor	Case-patients, n = 14	Controls, n = 22	OR or SD (95% CI)
Employment status			
Employed	7 (50)	12 (55)	0.8 (0.2–3.1)
Retired	2 (14)	4 (18)	0.7 (0.1–4.2)
Student or dependent child	5 (36)	5 (23)	1.9 (0.4–7.9)
Occupational outdoor activities	5/7 (71)	4/12 (33)	5.0 (0.7–33.5)
More outdoor recreation because of COVID-19 pandemic	6 (43)	10 (45)	0.9 (0.2–3.4)
Recreational outdoor activities			
>4 h outside per weekday	6 (43)	5 (23)	2.5 (0.6–10.5)
>4 h outside per weekend day	12 (86)	11 (50)	**6.0 (1.2–29.2)**
Average total time outside, h (SD)	83.3 (77.2)	51.3 [47.5)	31.9 (–16.1 to 80.0)
Specific recreational outdoor activities			
Yard work or gardening	8 (58)	15 (68)	0.6 (0.2 to 2.4)
Average no. times (SD)	2.9 (1.7)	7 [4.7)	**–4.0 (–6.8 to –1.2)**
Hunting or fishing	5 (36)	7 (32)	1.2 (0.3 to 4.7)
Average no. times (SD)	2.8 (1.6)	3.1 [1.0)	–0.3 (–2.4 to 1.7)
Hiking/walking/running on unpaved trails	12 (86)	15 (68)	2.8 (0.5 to 13.9)
Average no. times (SD)	5.6 (3.5)	8.9 [6.0)	–3.3 (–7.2 to 0.7)
Camping	6 (43)	6 (27)	2.0 (0.5 to 8.0)
Average no. times (SD)	4.2 (1.7)	2.8 [1.2)	1.3 (–0.6 to 3.3)
Off-road mountain biking	2 (14)	5 (23)	0.6 (0.1 to 3.1)
Average no. (SD)	5 (0)	3.3 [2.2)	1.7 (–1.0 to 4.4)
Personal tick prevention measures			
Used repellent or repellent-treated clothing	3 (21)	10 (45)	0.3 (0.1–1.4)
Checked self for ticks	14 (100)	10 (45)	**34.5 (3.0–365.3)**
Wore long pants	11 (78)	15 (68)	1.7 (0.4–7.5)
Wore long sleeves	12 (86)	14 (64)	3.4 (0.7–16.9)
Found a tick on self	14 (100)	9 (41)	**41.2 (3.5–436.6)**
Found tick attached to self	13 (93)	8 (36)	**22.7 (3.0–156.0)**
Location where tick acquired			
National Forest	8/12 (67)	4/4 (100)	0.2 (0.0–3.0)
State Park	2/12 (17)	0/4 (0)	2.1 (0.1–27.0)
Private land	2/12 (17)	0/4 (0)	2.1 (0.1–27.0)

*Values are no (%) except as indicated. Bold indicates statistical significance (α = 0.05). OR, odds ratio.

**Figure 2:**
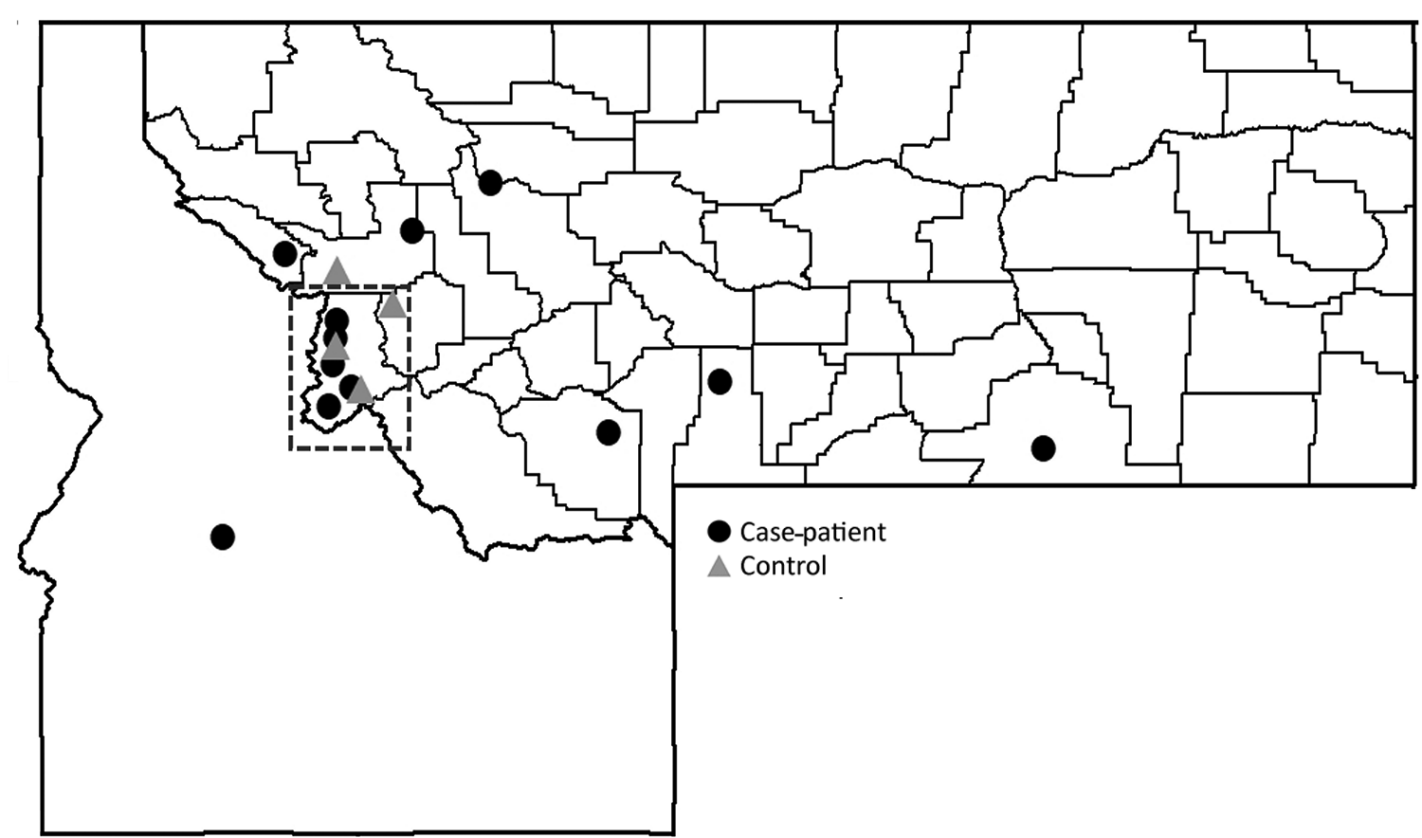
Geographic locations in Montana and Idaho, USA, where case-patients (n = 12) and controls (n = 4) reported tick acquisition during the 2 weeks before symptom onset in a Colorado tick fever case–control study, Montana, USA, 2020. Ravalli County (dashed box) had the most case-patient tick acquisitions (5); Missoula, Mineral, Madison, Park, Big Horn, and Lewis and Clark each had 1 acquisition. One case-patient acquired a tick in Idaho.

Among patients who reported finding ticks on themselves, 86% (12/14) of case-patients and 44% (4/9) of controls provided specific information about where they acquired the tick or ticks (Figure 2). Tick exposures occurred in national forests, in state parks, and on private land in several areas of Montana; 6/12 case-patients reported acquiring their tick or ticks in the vicinity of the Bitterroot Valley (5 in Ravalli County and 1 in Mineral County). One Montana resident with CTF reported acquiring a tick in Idaho (Figure 2). Except for 1 case-patient, all other case-patients reported that their tick exposures occurred in locations >4,000-ft elevation.

### Healthcare Provider Survey

A total of 36 healthcare providers from 10 public health jurisdictions responded to the survey. Most respondents were physicians (22 [61%]), worked in outpatient clinics (24 [67%]), and had a nonpediatric specialty (26 [72%]). The median number of years practicing was 15 years (range 1–41 years), including 10 years (range 0.5–40 years) practicing in Montana. Only 2 providers indicated that they had more patients reporting tick bites in 2020 than in 2019, whereas 5 had fewer patients reporting tick bites in 2020 than in 2019. Three healthcare providers reported ordering more CTF virus tests in 2020 than in 2019, and 2 providers ordered fewer CTF tests. Most providers (31 [86%]) reported no change in their awareness of CTF virus in 2020 compared with 2019. Many providers (26 [72%]) were interested in educational resources for CTF.

### Laboratory Testing Evaluation

Before 2020, testing for CTF virus in Montana was conducted by the state public health laboratory using an indirect immunofluorescence assay (IFA) to detect CTF virus–specific IgG antibodies (*16*,*17*). Cases were reported as probable if virus-specific IgG was detected in a single serum specimen and confirmed if a 4-fold rise in IgG was detected between paired acute and convalescent serum specimens. During 2011–2019, a median of 156 specimens (range 90–208 specimens) were tested for CTF annually by using IFA, and average positivity was 1.3% (Table 4). We did not have data on the numbers of specimens tested per patient or timing of specimen collection in relation to onset.

**Table 4:** Laboratory testing for Colorado tick fever virus infection performed on specimens from Montana, USA, 2011–2020*

Year	Assay	No. tests performed	No. (%) tests positive
2011	IFA	90	0
2012	IFA	141	2 (1.4)
2013	IFA	168	0
2014	IFA	143	2 (1.4)
2015	IFA	199	4 (2.0)
2016	IFA	120	3 (2.5)
2017	IFA	172	4 (2.3)
2018	IFA	208	3 (1.4)
2019	IFA	170	3 (1.8)
2020	RT-PCR	84	18 (21.4)
	PRNT	53	3 (5.7)
	Total	137	21 (15.3)

*IFA, indirect immunofluorescence assay for IgG; PRNT, plaque-reduction neutralization test; RT-PCR, reverse transcription PCR.

In 2020, because of the surge in SARS-CoV-2 testing, Montana began sending specimens for CTF testing to CDC, which primarily used RT-PCR for acute specimens and PRNT for convalescent specimens. A total of 137 CTF tests were performed at CDC on specimens sent on behalf of 107 Montana residents with symptom onset in 2020; of those, 21 tests (15.3%) were positive. Of the RT-PCR tests performed, 18/84 (21.4%) were positive (confirmed cases) on serum specimens collected a median of 3 days (range 0–14 days) after symptom onset. Of the PRNT tests performed, 3/53 (5.7%) were positive (probable cases) on serum specimens collected 17, 23, and 42 days after symptom onset. For 25 patients, RT-PCR and PRNT were performed on the same specimen; of these, 4 had discordant results, 3 (12%) were RT-PCR-positive and PRNT-negative (collected 8, 12, and 14 days after onset), 1 was RT-PCR-negative and PRNT-positive (collected 17 days after onset), and 21 had concordant negative results (Table 4).

During 2020, no changes occurred in testing for RMSF or tularemia. The numbers of cases reported were within the range of those reported during previous years in Montana: 2 cases of RMSF reported in 2020 compared with an average of 5 cases (range 1–11 cases) during 2010–2019, and 1 case of tularemia in 2020 compared with an average of 4 cases (range 1–7 cases) during 2010–2019.

## Discussion

Using a multifaceted approach, we investigated the effect the COVID-19 pandemic might have had on CTF virus infection risk and diagnosis in Montana in 2020. Because of the pandemic, persons were more likely to spend time outdoors, and many persons with CTF reported acquiring ticks in areas of Montana known to be endemic for CTF virus. However, a switch from serologic to primarily molecular testing on acute-phase specimens that occurred because of the surge in demand for SARS-CoV-2 testing was most likely responsible for the increase in CTF cases detected.

Unlike most domestic arthropodborne viruses, CTF virus infection is characterized by a sustained viremic period caused by infection of hematopoietic progenitor cells and delayed antibody response (*12*). CTF viral RNA can usually be detected by RT-PCR during the first 2 weeks of illness and for up to 6 weeks after illness onset (*1*,*7*,*12*). CTF virus–specific antibodies are undetectable in >50% of patients at 2 weeks after illness onset, but neutralizing antibodies are detectable in >90% of patients by 4 weeks after illness onset (*1*,*7*,*11*,*16*). Therefore, RT-PCR testing is recommended for acute-phase specimens (collected <14 days after symptom onset), and serologic testing (e.g., IFA or PRNT) is recommended for specimens collected >2 weeks symptom onset (*11*,*12*,*18*). Previous studies comparing IFA to PRNT assays for serologic diagnosis of CTF demonstrated comparable performance (*16*). As a crude comparison, we found that the proportion of RT-PCR tests positive during 2020 was ≈20-fold higher compared with the proportion of tests positive by IFA in previous years; however, we were unable to test specimens from previous years to directly compare the assay performances.

CTF used to be one of the most frequently reported domestic arboviral diseases. Beginning in the late 1980s, the numbers of CTF cases reported to CDC began decreasing, probably because of changes in land use, testing, and reporting practices (*4*,*7*). Because CTF is not a nationally notifiable condition, reporting is based on individual state requirements, and not all states where CTF virus is known to be endemic have reported consistently over time (*7*). Furthermore, diagnostic testing methods among states are variable; before 2006, most available CTF testing was done by serologic testing only (*4*,*7*). Therefore, historical trends are difficult to assess, and the actual prevalence of CTF is probably underappreciated.

The findings of this investigation are consistent with national trends showing that outdoor recreation increased during the COVID-19 pandemic. More time spent recreating outdoors can increase the likelihood of persons being exposed to vectors that can transmit infections. A recent report on Lyme disease surveillance in the United States found that ≈50% of respondents to a consumer survey reported spending more time outdoors in 2020 than in previous years, similar to our results (*19*). Reports from Switzerland and Germany during the summer of 2020 demonstrated a substantial increase in endemic tickborne encephalitis virus but decreases in travel-related vectorborne diseases such as dengue and malaria (*20*,*21*). Both reports attributed the increase in cases primarily to increased outdoor activity. Although 44% of our participants reported spending more time outdoors in 2020 compared with 2019, case-patients and controls reported a similar change in behavior. Furthermore, we did not see an increase in the number of other diseases transmitted by the same tick vector for which testing algorithms did not change, including RMSF and tularemia, and providers did not report an increase in patient encounters with tick exposure histories in 2020. Although tularemia can be spread by other routes, the lack of an increase in either of these diseases is supportive evidence that the CTF testing protocol change was the major factor leading to an increase in CTF cases detected.

These findings also indicate that the locations where case-patients spent their time recreating and were exposed to ticks probably contributed to the case-patients becoming infected. In addition, greater awareness of tickborne diseases in highly endemic areas might have led to more testing of patients exposed in these regions. Half (6 of 12) of patients with CTF who knew where they acquired a tick before becoming ill reported acquiring ticks in the Bitterroot Valley. The Bitterroot Valley, located between the Bitterroot and Sapphire Mountains in Ravalli and Missoula Counties, has historically been an endemic area for CTF virus; a survey of ticks collected during 2002–2003 and 2009–2013 in the Bitterroot Valley found a 6.6% prevalence of infection with CTF virus (*22*). On the basis of the specific location data provided by participants, CDC and the Montana Department of Public Health and Human Services worked with partners at the US Forest Service and Montana Department of Fish, Wildlife, and Parks to distribute social media messages and trail signs on tick-prevention measures and CTF awareness to targeted areas identified during this investigation.

The first limitation of our study is that the small number of participants and our convenience sample of controls with suspected tickborne diseases could have limited the precision of our estimates and our power to detect differences. In addition, given the relatively few case-patients able to participate in the study, reliable evaluation of confounding or identification of potential effect modifiers of the identified risk factors was not possible. We report a range of 95% CI estimates for both ORs and mean differences, and several of these are wide, given the context of the application. Although our results are consistent with what is known about the epidemiology of CTF in Montana and are not unexpected, caution should be used when interpreting imprecise results. Further, selection was nonrandom, and participation was self-selected, so our results may be subject to related unmeasurable biases. The results were probably subject to participant recall bias and accentuated by delays in the investigation related to the COVID-19 pandemic. Finally, although the molecular testing performed at CDC might have increased the proportion of specimens identified as positive, we did not test specimens from previous years to directly compare sensitivities of the assays.

To help potentially improve case detection and to address healthcare provider interest in additional CTF resources identified during this investigation, CDC developed and distributed a pocket card with information about CTF, including epidemiology, clinical findings, prevention, and testing recommendations (https://www.cdc.gov/coloradotickfever/diagnostic-testing.html). CDC also created a tickborne viral disease training module for clinicians, which includes information on CTF, Powassan, Heartland, and Bourbon virus diseases and provides continuing education credits (https://tceols.cdc.gov/course/detail2/8642).

In summary, the increase in CTF cases reported by Montana in 2020 was most likely caused by the shift in use of molecular testing for CTF, which is recommended during the acute phase of illness. State public health laboratories should consider molecular testing for CTF, and support for adopting this testing platform can be obtained from CDC on request. Greater outdoor recreation, particularly in areas endemic for CTF, was an identified modifiable risk factor. Although outdoor recreation should be encouraged, persons who work or recreate in CTF-endemic areas should continue to be advised to take precautions to avoid tick exposures, such as using insect repellent approved by the US Environmental Protection Agency, performing tick checks when returning from outdoor activities (https://www.cdc.gov/ticks/avoid/on_people.html), and promptly removing ticks.
